# De-escalation strategies for non-pharmaceutical interventions following infectious disease outbreaks: a rapid review and a proposed dynamic de-escalation framework

**DOI:** 10.1186/s12992-021-00743-y

**Published:** 2021-09-16

**Authors:** Charbel El Bcheraoui, Sophie Alice Müller, Eleanor C Vaughan, Andreas Jansen, Robert Cook, Johanna Hanefeld

**Affiliations:** 1 Evidence-Based Public Health, Centre for International Health Protection, Robert Koch Institute, Nordufer 20, 13353 Berlin, Germany; 2 Centre for International Health Protection, Robert Koch Institute, Nordufer 20, 13353 Berlin, Germany; 3 The Economist Intelligence Unit, 20 Cabot Square, E14 4QW London, UK; 4 Information Centre for International Health Protection, Centre for International Health Protection, Robert Koch Institute, Nordufer 20, 13353 Berlin, Germany

**Keywords:** Outbreaks, Health system, COVID-19, Non-pharmaceutical interventions, Health surveillance, De-escalation strategies, Health policy

## Abstract

**Background:**

The severity of COVID-19, as well as the speed and scale of its spread, has posed a global challenge. Countries around the world have implemented stringent non-pharmaceutical interventions (NPI) to control transmission and prevent health systems from being overwhelmed. These NPI have had profound negative social and economic impacts. With the timeline to worldwide vaccine roll-out being uncertain, governments need to consider to what extent they need to implement and how to de-escalate these NPI. This rapid review collates de-escalation criteria reported in the literature to provide a guide to criteria that could be used as part of de-escalation strategies globally.

**Methods:**

We reviewed literature published since 2000 relating to pandemics and infectious disease outbreaks. The searches included Embase.com (includes Embase and Medline), LitCovid, grey literature searching, reference harvesting and citation tracking. Over 1,700 documents were reviewed, with 39 documents reporting de-escalation criteria included in the final analysis. Concepts retrieved through a thematic analysis of the included documents were interlinked to build a conceptual dynamic de-escalation framework.

**Results:**

We identified 52 de-escalation criteria, the most common of which were clustered under surveillance (cited by 43 documents, 10 criteria e.g. ability to actively monitor confirmed cases and contact tracing), health system capacity (cited by 30 documents, 11 criteria, e.g. ability to treat all patients within normal capacity) and epidemiology (cited by 28 documents, 7 criteria, e.g. number or changes in case numbers).

De-escalation is a gradual and bi-directional process, and resurgence of infections or emergence of variants of concerns can lead to partial or full re-escalation(s) of response and control measures in place. Hence, it is crucial to rely on a robust public health surveillance system.

**Conclusions:**

This rapid review focusing on de-escalation within the context of COVID-19 provides a conceptual framework and a guide to criteria that countries can use to formulate de-escalation plans.

**Supplementary Information:**

The online version contains supplementary material available at 10.1186/s12992-021-00743-y.

## Introduction

As of early June 2021, there have been over 170 m cases globally and over 3.7 m deaths attributed to COVID-19, the disease caused by a novel strain of the coronavirus. [1] On February 3rd 2020 the World Health Organization (WHO) published a Strategic preparedness and response plan for COVID-19, based on mitigating the pandemic by limiting human-to-human transmission: this is done by reducing international spread, reducing social contacts and isolating infected patients. [2] The two key strategies in pandemic control are mitigation and suppression. [3] Mitigation centres on the concept of “flattening the curve”, where the overall number of cases is distributed over a greater period of time, allowing the health system to respond within its capacity. [3, 4] Suppression aims to reduce the number of cases through the use of intensive non-pharmaceutical interventions (NPI) such as physical distancing and school closures until effective countermeasures – either treatment or vaccine – are available. [5] At the start of the pandemic, some countries began by introducing mitigation measures, while most moved to an approach that blends mitigation and suppression. [3] A limited number of countries such as Australia, followed a CVID-Zero approach through complete shutdown for a period of time, followed by almost-complete borders closure. [6] Introducing such strict measures raises ethical, social, political, economic and legal issues that must be considered to ensure their feasibility and sustainability. [7, 8]

Physical distancing is the “first line of defence” against outbreaks of novel strains for which there is not yet a vaccine or effective treatment, when it is activated without delay and maintained for a relatively long period. [9, 10] While a number of vaccines effective against COVID-19 have been developed, approved, and are being rolled out, there remains huge uncertainty around the timeline for a worldwide effective vaccination coverage, with physical distancing in place for anywhere between 25 and 70 % of that time. [3, 11, 12] The strict lockdown measures during this pandemic come with inherent social and economic consequences. In some places and industries, lockdown has led to a virtual shutdown, increasing the financial burden on governments to support people through welfare (unemployment and furlough support), as well as seeing surges in interpersonal violence and potential mental health impacts. [13–18] An indefinite timeline for a return to normal or a “new normal” puts public compliance at risk as strict NPI become socially, politically and economically unacceptable and unviable. [3, 8, 12, 19, 20] However, we have seen two additional major threats during the COVID-19 pandemic: first, there is a risk of resurgence when interventions are de-escalated, and second, the unanimity of control measures and public health interventions has led to the emergence of more transmissible variants of concerns, including some that are vaccine-escaping, from the original SARS-CoV-2wildtype. [3, 4, 11, 21] This creates an imperative for governments to use transparent criteria that allow NPI to be de-escalated and re-escalated in response to the changing epidemiological situation.

Thresholds for signalling the start and end of a pandemic and for escalating response measures within a pandemic are frequently described in national pandemic planning documentation. [22, 23] However, criteria for de-escalating responses are less clear – aside from the implicit suggestion that they are the opposite of the escalation criteria. Hence, we reviewed the literature to identify de-escalation criteria and developed a conceptual framework that policy makers could follow to plan their de-escalation strategies through the continued, as well as future pandemics.

## Methods

The scope of this review was to identify documents relating to the de-escalation of interventions introduced in response to, or included in planning documents for pandemics or infectious disease outbreaks, published from 2000 onwards. No geographic or language limits were applied.

### Searching

An initial search took place in April 2020 and focused on combining terms around the key concepts – infectious disease outbreaks/pandemics and de-escalation. Search terms per database are detailed in Additional file 1 pp 1–3. Initially the plan was to use an all-hazards approach to investigate triggers for de-escalation in the broader disaster-preparedness literature. However, scoping revealed that this approach retrieved a large volume of material with limited applicability to infectious disease pandemics (for example, physical rebuilding following earthquakes).

The main issue was that no single term is consistently used in the literature to refer to the concept of “de-escalation”. Indeed, we identified over 30 distinct terms used to describe de-escalation, with different terminology used within the same documents. The terms that are used to describe this concept are also not unique to public health or emergency preparedness, so it was challenging to design a search strategy that balances sensitivity and precision. For the purposes of this report, we selected the term “de-escalation” as it is used by the European Centre for Disease Prevention and Control (ECDC). [23, 24]

The Initial scoping revealed that bibliographic databases were unlikely to yield the documents relevant to the review because of a lack of standard language and appropriate thesaurus headings. Hence, we approached the literature from different angles, using different approaches and a range of sources (Table 1). We repeated the search in June 2021. This time, the search yielded a higher number of peer-reviewed manuscripts related to the topic, and COVID-19-related, and hence no search of the grey literature was preformed again for the period of April 2020 – June 2021.

**Table 1:** Search process of the literature on de-escalation strategies of non-pharmaceutical interventions following an infectious disease outbreak

1. Grey literature searches to include unindexed sources of information from institutional websites such as the US Centers for Disease Control and Prevention (CDC), the European Centre for Disease Prevention and Control (ECDC) and WHO [25]
2. Advanced searching techniques such as citation tracking and reference harvesting from key documents
3. Searches in the specialist COVID-19 source LitCovid [26]
4. Searches in Embase.com (which includes Embase and Medline) bibliographic database (covering the biomedical, public health and disaster literature) on April 8th 2020 and Scopus
5. Review of the list of rapid reviews prepared externally for WHO (unpublished, updated April 9th 2020)
6. The University of Oxford Coronavirus Government Response Tracker [27]

### Sifting

Literature retrieved from bibliographic databases was sifted first based on title and abstract, then based on abstract and/or full text, based on the criteria below:

- Title/abstract sift, documents were included if they met the following criteria:
  - Date limits – from 2000.
  - Event type – pandemic or infectious disease outbreak.
- Abstract/full text sift, documents were included if they met the following criteria:
  - Cross-border/international infectious disease outbreak.
  - Describe the national response to an international infectious disease outbreak.
  - Included reference to de-escalation.
- At the data extraction stage, documents were included if they met the following criteria:
  - Reported de-escalation criteria.

### Analysis

Data analysis began with a standardised data extraction form created for this project, capturing the following information:

- Setting – country, year, health system type.
- Type of hazard, risk, emergency or event.
- The de-escalation decision-making process – for example, what triggers were used, thresholds reached etc.
- The de-escalation process – what measures/interventions were removed? When and how?
- Outcomes of de-escalation – was de-escalation deemed “successful”? Was there a need for the reintroduction of de-escalated interventions?

### Framework development

We followed the method proposed by Jabareen in 2009 to develop a conceptual framework for de-escalation of NPI following infectious disease outbreaks. [28] A conceptual framework provides an interpretative approach – hence an understanding, instead of a theoretical explanation – to social reality. Concepts, represented in boxes, were retrieved from the thematic analysis of the included documents and interlinked based on the components originating from the previous or the following concepts in a logical sequence.

## Results

**Fig. 1:**
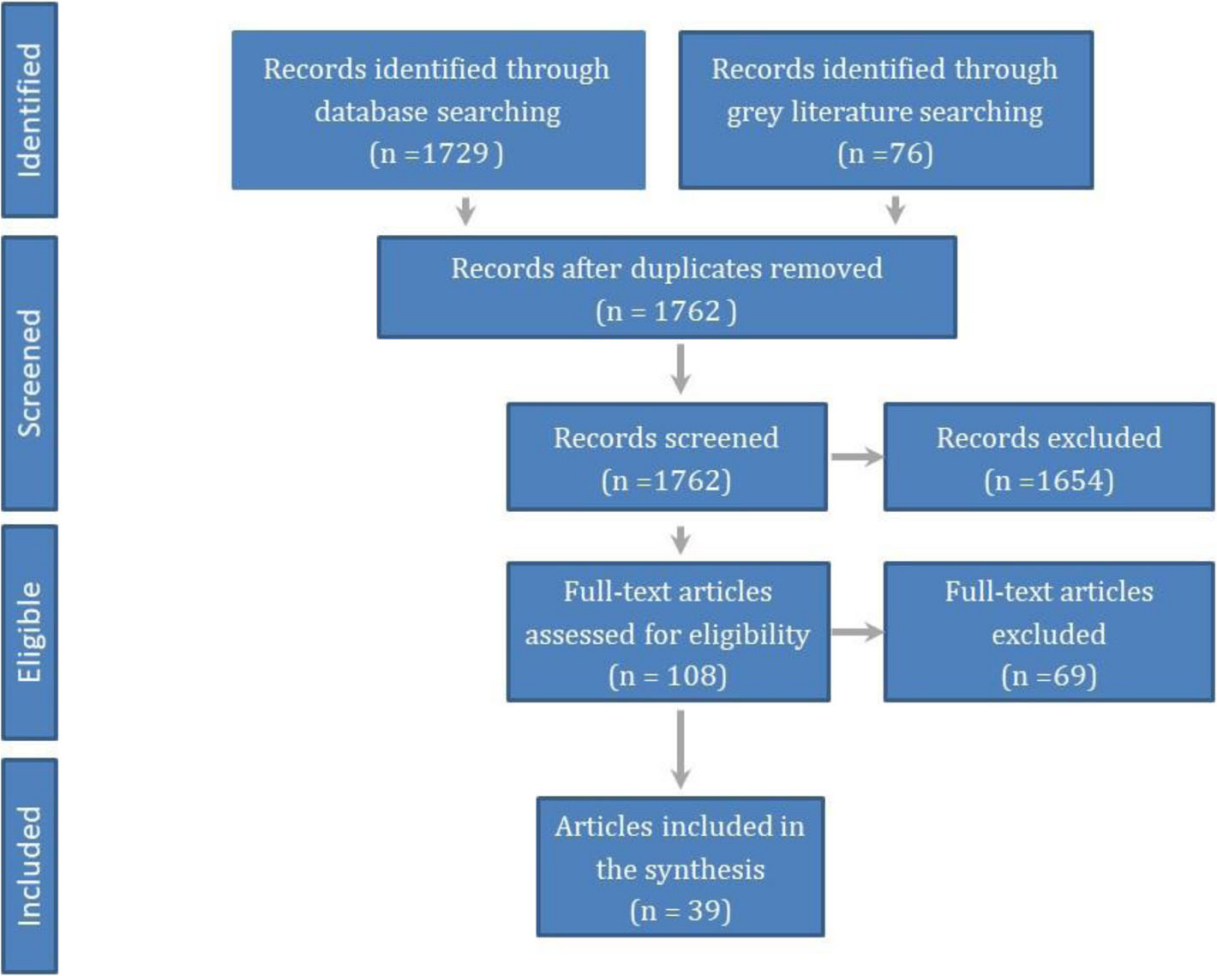
PRISMA flow diagram of the review of de-escalation of non-pharmaceutical interventions following infectious disease outbreaks for the searches of April 2020 and June 2021 combined

Of a total of 1,762 documents identified, 39 reported de-escalation criteria and were hence included in the analysis. Figure 1 details the identified, screened, eligible and analysed documents. The 39 fully analysed documents are listed in Additional file 2 pp 1, clustered by the type of document. In these documents, de-escalation was described as selective, phased or adaptive. Selective de-escalation is selective based on either demography or geography – identifying groups of people or areas that are high- or low-risk, so that interventions can be de-escalated for those in the low-risk categories. Phased de-escalation refers to a process that is graduated, so interventions are partially or fully de-escalated and not all at once, with continued to monitoring to ensure that there is no adverse effect on case numbers.

**Fig. 2:**
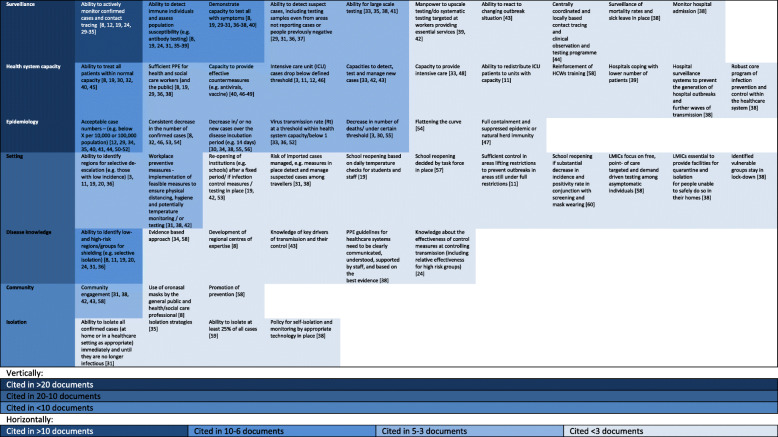
Heat map of criteria suggested for de-escalation of non-pharmaceutical interventions following infectious disease outbreaks

In total we identified 52 criteria recommended to consider for de-escalating NPIs (Fig. 2). We clustered under the surveillance system capacity, health system capacity, isolation capacity, disease epidemiology, disease knowledge, variability in settings, and community engagement/behaviour.

### Most reported surveillance, health system capacity and epidemiology -based criteria

The analysis of documents reporting de-escalation criteria highlighted a range of criteria based on epidemiology, disease knowledge, and healthcare, surveillance, setting-specific, isolation and community capacity. The most commonly reported categories of de-escalation criteria were surveillance, health system capacity and epidemiology-based criteria.

Ten criteria were grouped as surveillance-based de-escalation criteria and cited by 43 documents. These criteria focused on the ability to monitor confirmed cases and contact tracing, [8, 12, 19, 24, 29–35] the assessment of susceptibility in a population, [8, 19, 24, 31, 35–39] as well as on capacity of testing all with symptoms, [8, 19, 29–31, 36–38, 40] the ability for large scale testing, [33, 35, 38, 41] or the ability to detect suspect cases. [29, 31, 36, 37] Less often cited criteria were the upscaling of manpower for testing, [39, 42] the ability to react to a changing outbreak situation, [43] the centrally coordinated contact tracing [44] or the surveillance of mortality rates [38] and hospital admissions. [38]

In terms of health system capacity, 30 documents named one or more of the eleven de-escalation criteria. Within these health system capacity criteria, the ability to treat all patients within normal capacity was the most commonly reported criteria, [8, 19, 30, 32, 40, 45] followed by sufficient PPE for health/social workers and/or the public [8, 19, 29, 36, 38] and the capacity to provide effective countermeasures such as antivirals and vaccines. [40, 46–49] Intensive care unit (ICU) capacity was also a specific consideration with the reported criteria, such as ICU cases dropping below a defined threshold and the ability to redistribute ICU patients between units to manage capacity. [3, 11, 12, 46] One study also named hospital surveillance systems to prevent the generation of hospital outbreaks and robust core program of infection prevention and control. [38]

Seven epidemiology-based de-escalation criteria were found and cited by 28 documents. These criteria focused on achieving a threshold of case numbers that were reported as “acceptable” in the local context; in some cases, specific numbers per 10,000 or 100,000 were reported, but this was not consistent. [12, 29, 34, 35, 40, 41, 44, 50–52] Observing a “consistent” decrease in the number of confirmed cases was also a commonly reported criteria, but specific thresholds were not reported. [8, 32, 46, 53, 54] Other criteria included decreases in the number of new cases over the disease incubation period [30, 34, 38, 55, 56] and an overall decrease in the number of deaths; again, thresholds were not specified. [3, 30, 55] Three documents reported using virus transmission rates (Rt) as a criteria [33, 35, 52] and one using full containment and suppressed epidemic or natural herd immunity. [47]

### Setting-, disease knowledge-, community- and isolation-based criteria

The ability to identify regions for selective de-escalation based on geography was reported within setting-specific capacity. [3, 11, 19, 20, 36] For the re-opening of workplaces, the implementation of feasible measures in line with the principles of the current non-pharmaceutical interventions in place were recommended by the WHO and others. [31, 38, 42] Other setting-specific criteria included those referring to re-opening schools either after a fixed period (a modelling simulation study) or when infection control, testing, task force or temperature check measures are in place. [19, 42, 53, 57] Managing the risk of imported cases during de-escalation by ensuring that sufficient measures are in place to detect and manage suspect cases among travellers was also reported. [31, 38] Ensuring that transmission control in areas that are de-escalating remains sufficient to prevent transmission to areas still under full restrictions was also reported as a criteria to consider in geography-based selective de-escalation. [11] Low and middle-income countries should focus on criteria such as free point- of care targeted and demand driven testing among asymptomatic individuals [58] and the provision of facilities for quarantine and isolation for people unable to safely do so in their homes. [38]

Disease knowledge-based criteria focused on the ability to identify high- and low-risk regions or groups for selective de-escalation based on demography. [8, 11, 19, 20, 24, 31, 36] Two manuscripts specifically focused on an evidence-based approach for de-escalation. [34, 58] Knowledge about the effectiveness of control measures, including their relative effectiveness for high-risk groups, was also a criterion for de-escalation. [24] Additional criteria were the knowledge of key drivers for transmission and their control. [43] The establishemnt of regional centres of expertise to share knowledge was reported as an important approach to inform de-escalation. [8]

Community-based criteria included engagement of communities to ensure understanding on how to contribute to the de-escalation process [31, 38, 42, 43, 58], as an important component of pandemic response. One paper also recommended that the use of facial masks be rolled out to the general public [31] and that prevention in general should be promoted within communities. [58]

The WHO also specifically discussed isolation capacity, in terms of ensuring that countries have the ability to isolate all confirmed cases and their close contacts, whether in their homes or healthcare settings. [31] One other study underlined the ability to isolate at least 25% of all cases. [59]

#### Process and outcomes of de-escalation of non-pharmaceutical interventions

Three documents specified details of the process of de-escalation. The European Centre for Disease Prevention and Control (ECDC) states that countries should assess whether surveillance and monitoring is robust enough to detect any possible infection resurgence before considering de-escalating any non-pharmaceutical interventions. [24] One document specified that the “re-opening of society should be staged according to the local situation”, and further detailed a four-stage approach of gradual re-opening. [38] The gradual re-opening steps were dependent on the observation of hospital admissions and performance of testing of all symptomatic persons for two weeks.

The Australian Department of Health described key activities within the de-escalation process: [45]

- Support and maintain quality care.
- Cease response interventions that are no longer needed.
- Transition response interventions to seasonal or interim arrangements.
- Monitor for a second wave of the outbreak.
- Monitor for the development of antiviral resistance.
- Communicate to support the return from pandemic to normal business services.
- Evaluate systems and revise plans and procedures.

Our review also sought to identify evidence on the outcomes of de-escalation of interventions, for example on the number of cases. Such information – beyond a theoretical risk of unquantified “resurgence” – was close to null in the over 1,700 documents reviewed. Only one observational study in Israel showed a gradual increase of the SARS-CoV-2 incidence following the re-opening of schools without an increase in COVID-19 associated hospitalizations and deaths. [60] Another example from Hong Kong reported an abrupt community outbreak after lifting restrictions on physical distancing and the reopening of schools. [56] One document – a theoretical de-escalation plan – stated that a sustained rise in new cases over five days or the exceeding of hospital capacity could be outcomes of de-escalation that trigger the re-escalation of non-pharmaceutical interventions. [30]

#### Proposed ***Dynamic de-escalation framework***

**Fig. 3:**
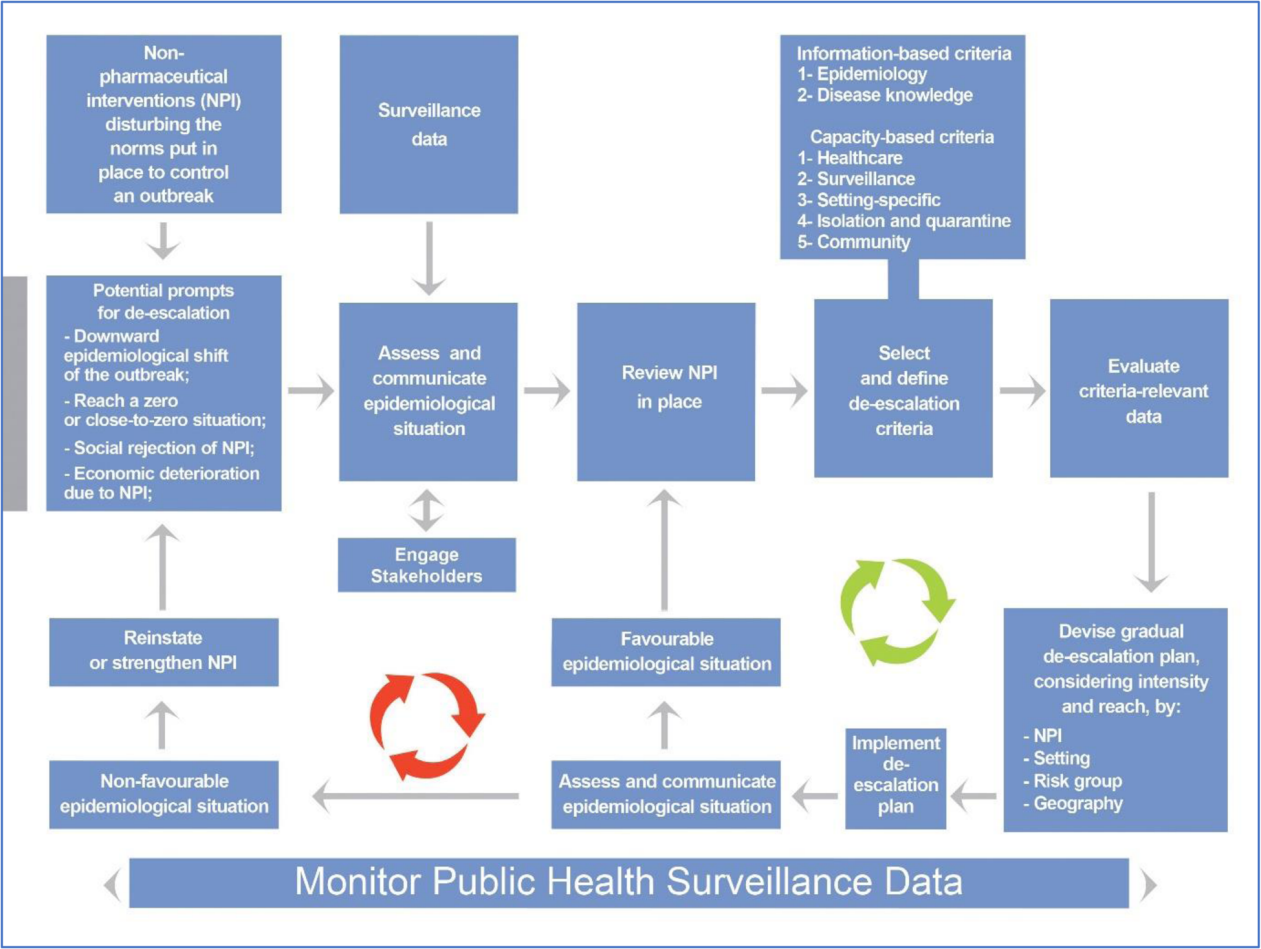
Dynamic de-escalation framework for non-pharmaceutical interventions following infectious disease outbreaks

The proposed Dynamic de-escalation framework is represented in Fig. 3. It is designed to provide a guide to government and public health agencies on how to de-escalate NPI. The framework provides a transparent process with clear criteria that aims to balance epidemiological, social, political and economic consideration. Decision and review points are included throughout to enable a dynamic process that can respond to changing circumstances as an outbreak progresses and provide a response that is appropriate to the current situation.

### Start of the de-escalation process

The start of the process defined by the framework can be prompted by the downward epidemiological shift of the outbreak, reaching a zero- or close-to-zero-case situation, the social rejection of NPI, or the economic deterioration due to the NPI. Community engagement is one of the key pillars of health emergency response and fundamental for the successful implementation of response measures by ensuring that communities are informed and equipped to play their part. [31] Hence, before moving to decision-making, it is important that key stakeholders, specifically those that work closely with their communities such as grassroot or community-based organisations, are engaged to ensure that all perspectives are considered in selecting and prioritising de-escalation criteria. [61] Following a participatory approach to decision-making can ensure buy-in and increase adherence to decisions taken. Once the decision has been made to transition to de-escalation, the next step is to assess and communicate the epidemiological situation. Well-thought risk communication strategies should deliver concise, useful, and appropriate messaging relative not only to the epidemiological situation, but also to protective measures and social responsibility. At this point, the appropriate stakeholders need to map and review the NPIs currently in place.

### Designing a de-escalation strategy

Once NPIs are mapped and reviewed, stakeholders can decide which criteria to use in determining which NPI to de-escalate, where, and at what pace. Specifically, stakeholders have to consider their original pandemic control model in terms whether they have chosen a “COVID-Zero” or a “living with COVID” approach. Considerations in terms of de-escalation would be different for these two approaches. For instance, countries that have chosen a COVID-Zero approach might place a higher emphasis on cross-country transmission and hence, border control measures, might be the last to de-escalate, if at all.

The reviewed literature points mostly to public health surveillance and health system capacity, as well as epidemiological criteria such as the ability to monitor confirmed cases and contacts, [8, 12, 19, 24, 29–35] the ability to treat all patients within normal capacity, [8, 19, 30, 32, 40, 45] or reaching an acceptable number of disease cases [12, 29, 34, 35, 40, 41, 44, 50–52] prior to de-escalating NPIs. When selecting NPIs for the de-escalation strategy, it is crucial to evaluate the data relative to chosen criteria. Each potential criterion should be carefully considered by assessing the sources of the data used to measure this criterion. When data sources for a criterion are not deemed reliable, it might be better to consider alternative criteria with more reliable data sources. Further, the selection of criteria and definition of thresholds should be guided by the dynamics of the pandemic, the social/economic context and the local outbreak situation within individual countries. Hence, we include criteria in the framework that reflect those in the literature, but we do not make recommendations or specifications on the selection and definition of de-escalation criteria using specific thresholds. To the extent possible, such thresholds should cover the possible outcomes of the decision-making process: what happens in case of no de-escalation, of selective de-escalation, of total de-escalation and of re-escalation.

Once criteria are selected, a gradual, rather than a one-time, approach of lifting NPIs is recommended. [38] This gradual approach gives the potential to be adaptive and pro-active with the changing outbreak situation while balancing health system capacity with data from surveillance systems. Epidemiological thresholds can thirdly contribute to a clear picture and classification of this balance. The strategy should consider which NPIs, and what pace should be de-escalated. The strategy should also be adapted to each specific setting, the potential risk groups, and the geographical areas. For instance, the de-escalation strategy in the case of COVID-19, cannot be similar between health care facilities responsible mainly for older persons in a high-incidence area, and schools in an area where incidence is close to zero.

### Implementation of a bi-directional de-escalation strategy

The implementation of the de-escalation strategy should be accompanied with a continuous monitoring and analysis of surveillance data together with the status of the health system capacity and epidemiological benchmarks to enable a permanent assessment of the situation – that is, whether a balance between these criteria has been reached, and how and whether de-escalation should proceed in a gradual process. If the epidemiological situation is stable or improved, further review of NPIs in place, and further consideration of de-escalation criteria and strategies can be considered. On the other hand, if the epidemiological situation is worsened or unstable, stakeholders should promptly consider re-instating some of all NPIs. Indeed, during this pandemic, we have seen at least two waves in any of the affected regions, in addition to the emergence of several variants of concerns proven less susceptible to the approved vaccines. [21]

The de-escalation decision-making process is hence not a one-time event – it will require review and revision as the situation evolves and circumstances change. The review of the de-escalation decision can be prompted by time, for example some governments have used two weeks as a review period, as it encompasses the disease incubation period [62] and emergence of symptoms to give a more accurate reflection of current case numbers. Prompts can also include changes in the routinely collected epidemiological data, such as increases or decreases in case numbers, or changes in healthcare utilisation (for example, increases or decreases in hospital or ICU admissions). Here again, community engagement along with a well-though risk communication strategy should be an integral part of this process.

## Discussion

We have screened over 1,700 documents at title and abstract or full text for de-escalation criteria for of NPI following infectious disease outbreak. Only 39 documents included de-escalation criteria for these NPI. This rapid review indicates that de-escalation is a bi-directional and gradual process whereby NPI are partially or fully de-escalated and can be re-escalated. Such a decision-making process should be informed throughout by robust public health surveillance data, balanced with social, political and economic considerations. Policy makers, along with their stakeholders, need to define context-relevant and socially-acceptable criteria to start the de-escalation process for each of the implemented NPI. Thresholds for each of the selected criteria have to also be contextually-defined and bi-directional. Throughout this process, decision-making should rely on a continued monitoring and use of multi-outcome surveillance data, paralleled with a constant evaluation of de-escalation effect indicators by intervention which should be defined as well.

The risk of subsequent wave(s) of epidemic infections cannot be removed entirely – unless countries opt to impose strict NPI until herd immunity is reached or entire populations are vaccinated. This is unfeasible as worldwide vaccine coverage only slowly increases while at the same time vaccine-escaping variants start appearing. Therefore, a key component of de-escalation planning becomes mitigating the risk of resurgence by ensuring that there is sufficient surveillance capacity to detect increases in the number of cases at an early stage, and health system capacity to cope and control the outbreak. [4, 8, 20, 24, 29, 31, 32, 45]

This review identified only one observational study that evaluated the impact or effectiveness of de-escalation, beyond the theoretical risk of resurgence. The retrieved evidence therefore is mainly based on mathematical models or grey literature. Notably, we found no evidence for a specific sequence of de-escalation of NPI, but only a proposed gradual four-stage de-escalation scheme. [38] Apart from this document, there was no evidence-based criteria to prioritize specific measures for early vs. late de-escalation. If such a list of priorities would exist, however, it would most likely be related to a specific cultural and societal background and thus not be generally applicable. While the sequence of de-escalation should be a result of an open societal discussion, it seems to be advisable to only suspend mitigation measures stepwise and gradually, so that the effects can be clearly attributed.

By the time this review was completed, the published peer-reviewed literature relevant to COVID-19 had not yet heavily focused on vaccine rollout. As vaccine roll-out is ongoing worldwide, we expect surveillance and health system capacity-related criteria to include monitoring of the population’s vaccination coverage as well as the ability to promptly distribute vaccines to the population.

### A dynamic, fit-for-purpose response

The fragile success of COVID-19 mitigation is constantly threatened by the risk of a subsequent wave(s) and the emergence of variants of concerns until a vaccine or effective antiviral treatment has been secured. Until then, an approach to de-escalation that responds to the changing social, economic and epidemiological situation, is needed. This is reflected in the *Dynamic de-escalation framework* devised based on this research.

Reviews of influenza pandemic preparedness plans and the response to the 2009 H1N1 flu pandemic highlighted the need for plans that are dynamic, as the consensus was that plans were based on a worst-case scenario and struggled to adapt to suit a less severe outbreak. [63–65] An unintended consequence of this reflection is that some countries have experienced difficulty in adapting plans to deal with the more serious scenario of COVID-19. The focus on influenza as the probable source of future pandemics may also have left the world inadequately prepared for COVID-19 when the disease did not follow the same dynamics as influenza. [19]

Reflecting on the COVID-19 and H1N1 experience, it is clear that pandemic planning needs to consider a variety of scenarios and epidemiological characteristics to enable a more agile response to future outbreaks. Including criteria for the escalation and de-escalation of outbreak response measures in pandemic plans enables their flexible implementation, with the timing, extent and use of interventions tailored to the severity of the outbreak at hand and factoring in other considerations such as social acceptability and the economic impact of NPI. [23, 65]

### Selective de-escalation

Selective de-escalation of interventions – either by geography (low-incidence areas), by social setting or demographic group (low-risk groups) – could enable countries to keep case numbers within a manageable number whilst balancing the health, social and economic impacts of NPI. This approach is predicated on sufficient knowledge about the disease to be able to identify high- and low-risk groups. [8, 11, 19, 20, 24, 31, 36] Testing capacity is also a pre-requisite to any de-escalation strategy to mitigate the risk of resurgence, something which many countries have struggled with. Selective de-escalation and re-escalation is an approach that is now being widely implemented.

It is difficult to assess the effectiveness of individual NPI, because they are usually deployed as a package of measures. [66] The timing of the introduction of NPI and measures contributes to the initial success and sustainability of these interventions, by ensuring community compliance. [8, 67] However, the basic truth that we cannot manage what we cannot measure implies that we need to evaluate whether NPI lead to the intended change. We don´t have this evidence for most of the COVID-19 mitigation measures.

This rapid review has some limitations. First, there is the possibility that this rapid review missed potentially relevant papers. We attempted to mitigate this risk through the use of a multi-faceted search approach not solely reliant on text-based searching. Standardising of terminology would improve the efficiency and accuracy of future research.

Second, while we did not limit the results to English language in our search, we used English language search terms only, meaning that the results would have been skewed towards English language documents. This was a pragmatic decision to enable a rapid review of the literature. On the opposite side, this rapid review included a wide range of documents, not limited to the scientific literature, this decision was taken in light of the rapidly evolving situation to enable the reviewers to consider emerging views on de-escalation of COVID-19 related NPI. Two researchers independently grouped all retrieved criteria in seven main domains. However, as there is no scientifically recognized framework, this grouping is not conclusively objective. Criteria such as “Risk of imported cases managed” could be either placed in surveillance but also in setting-specific criteria as the specify points of entry. Nevertheless, there remains room for discussion on developing additional domains or creating other sub-criteria.

## Conclusions

This rapid review of de-escalation criteria incorporated COVID-19 and previous infectious disease outbreaks and planning exercises. De-escalation is not a single activity, rather it is a bi-directional, dynamic process driven by changes in public health surveillance data that can lead to the partial or full re-escalation(s) of response and control measures. Our *Dynamic de-escalation framework* reflects this.

This rapid review has highlighted limited specific information or guidance for countries in selecting and defining de-escalation criteria, hence the need for the criteria and framework that we have devised. While escalation criteria – to define the start of a pandemic or within pandemic phases – are often reported, de-escalation criteria are rare beyond the inference that they are the opposite of the escalation criteria. Such criteria also require clear national definition informed by the evidence from epidemiological data and social and economic acceptability.

## Supplementary Information

**Additional file 1:**

**Additional file 2:**

## Data Availability

The datasets used and/or analyzed during the current study are available from the corresponding author on reasonable request.
