# Supplementary material for: De-escalation strategies for non-pharmaceutical interventions following infectious disease outbreaks: a rapid review and a proposed dynamic de-escalation framework
Source: Global Health. 2021 Sep 16;17:106. doi: 10.1186/s12992-021-00743-y (PMC8444163; doi:10.1186/s12992-021-00743-y)
Supplement: Supplementary file 1 — Additional file 1: [file 12992_2021_743_MOESM1_ESM.docx]

#### "This supplementary material is hosted by *Globalization and Health* as supporting information alongside the article [De-escalation strategies for non-pharmaceutical interventions following infectious disease outbreaks: a rapid review and a proposed dynamic de-escalation framework], on behalf of the authors, who remain responsible for the accuracy and appropriateness of the content. The same standards for ethics, copyright, attributions and permissions as for the article apply. Supplements are not edited by *Globalization and Health* and the journal is not responsible for the maintenance of any links or email addresses provided therein."

#### EMBASE search strategy

Table 1: EMBASE 1 search strategy

| **No.** | **Query** |
| --- | --- |
| #1 | 'public health'/exp/mj |
| #2 | 'epidemic'/exp/mj OR 'pandemic'/exp/mj |
| #3 | 'organization and management'/exp |
| #4 | #1 AND #2 AND #3 |

The second search, EMBASE 2, most closely resembled a big search. It combined all the key concepts; however, the output was relatively small, and on further testing the preparedness facet seemed not as well represented in relevant studies as we would have expected (hence EMBASE 3 search).

Table 2: EMBASE 2 search strategy

| **No.** | **Query** |
| --- | --- |
| #5 | 'public health'/exp/mj |
| #6 | 'public health':ti,ab |
| #7 | 'pandemic'/exp/mj OR 'epidemic'/exp/mj |
| #8 | pandemic:ti,ab OR epidemic:ti,ab |
| #9 | 'disease outbreak':ti,ab |
| #10 | 'public health'/exp/mj OR 'public health':ti,ab OR ('pandemic'/exp/mj OR 'epidemic'/exp/mj) OR (pandemic:ti,ab OR epidemic:ti,ab) OR 'disease outbreak':ti,ab |
| #11 | 'emergency preparedness'/exp OR 'preparedness'/exp OR 'disaster planning'/exp |
| #12 | (emergency NEAR/3 (prepar* OR respon* OR plan*)):ti,ab |
| #13 | (pandemic NEAR/3 (prepar* OR respon* OR plan*)):ti,ab |
| #14 | (outbreak NEAR/3 (prepar* OR respon* OR plan*)):ti,ab |
| #15 | 'after action review':ti,ab |
| #16 | 'critical incident review':ti,ab |
| #17 | 'organization and management'/exp/mj |
| #18 | ('emergency preparedness'/exp OR 'preparedness'/exp OR 'disaster planning'/exp) OR (emergency NEAR/3 (prepar* OR respon* OR plan*)):ti,ab OR (pandemic NEAR/3 (prepar* OR respon* OR plan*)):ti,ab OR (outbreak NEAR/3 (prepar* OR respon* OR plan*)):ti,ab OR 'after action review':ti,ab OR 'critical incident review':ti,ab OR 'organization and management'/exp/mj |
| #19 | 'exit strategy':ti,ab |
| #20 | 'de-escalat*':ti,ab OR 'de escalat*':ti,ab OR 'deescalat*':ti,ab |
| #21 | 'de-isolat*':ti,ab OR 'de isolat*':ti,ab OR deisolat*:ti,ab |
| #22 | 're-open*':ti,ab OR 're open*':ti,ab OR reopen*:ti,ab |
| #23 | (relax* NEAR/3 (restriction* OR measure* OR intervention*)):ti,ab |
| #24 | (lift* NEAR/3 (restriction* OR measure* OR intervention*)):ti,ab |
| #25 | (ease NEAR/3 (restriction* OR measure* OR intervention*)):ti,ab |
| #26 | (easing NEAR/3 (restriction* OR measure* OR intervention*)):ti,ab |
| #27 | 'trigger to lift':ti,ab OR 'triggers to lift':ti,ab OR 'trigger to ease':ti,ab OR 'triggers to ease':ti,ab OR 'trigger to relax':ti,ab OR 'triggers to relax':ti,ab OR 'threshold to lift':ti,ab OR 'thresholds to lift':ti,ab OR 'threshold to ease':ti,ab OR 'thresholds to ease':ti,ab OR 'threshold to relax':ti,ab OR 'thresholds to relax':ti,ab |
| #28 | 'exit strategy':ti,ab OR ('de-escalat*':ti,ab OR 'de escalat*':ti,ab OR 'deescalat*':ti,ab) OR ('de-isolat*':ti,ab OR 'de isolat*':ti,ab OR deisolat*:ti,ab) OR ('re-open*':ti,ab OR 're open*':ti,ab OR reopen*:ti,ab) OR (relax* NEAR/3 (restriction* OR measure* OR intervention*)):ti,ab OR (lift* NEAR/3 (restriction* OR measure* OR intervention*)):ti,ab OR (ease NEAR/3 (restriction* OR measure* OR intervention*)):ti,ab OR (easing NEAR/3 (restriction* OR measure* OR intervention*)):ti,ab OR ('trigger to lift':ti,ab OR 'triggers to lift':ti,ab OR 'trigger to ease':ti,ab OR 'triggers to ease':ti,ab OR 'trigger to relax':ti,ab OR 'triggers to relax':ti,ab OR 'threshold to lift':ti,ab OR 'thresholds to lift':ti,ab OR 'threshold to ease':ti,ab OR 'thresholds to ease':ti,ab OR 'threshold to relax':ti,ab OR 'thresholds to relax':ti,ab) |
| #29 | ('public health'/exp/mj OR 'public health':ti,ab OR ('pandemic'/exp/mj OR 'epidemic'/exp/mj) OR (pandemic:ti,ab OR epidemic:ti,ab) OR 'disease outbreak':ti,ab) AND (('emergency preparedness'/exp OR 'preparedness'/exp OR 'disaster planning'/exp) OR (emergency NEAR/3 (prepar* OR respon* OR plan*)):ti,ab OR (pandemic NEAR/3 (prepar* OR respon* OR plan*)):ti,ab OR (outbreak NEAR/3 (prepar* OR respon* OR plan*)):ti,ab OR 'after action review':ti,ab OR 'critical incident review':ti,ab OR 'organization and management'/exp/mj) AND ('exit strategy':ti,ab OR ('de-escalat*':ti,ab OR 'de escalat*':ti,ab OR 'deescalat*':ti,ab) OR ('de-isolat*':ti,ab OR 'de isolat*':ti,ab OR deisolat*:ti,ab) OR ('re-open*':ti,ab OR 're open*':ti,ab OR reopen*:ti,ab) OR (relax* NEAR/3 (restriction* OR measure* OR intervention*)):ti,ab OR (lift* NEAR/3 (restriction* OR measure* OR intervention*)):ti,ab OR (ease NEAR/3 (restriction* OR measure* OR intervention*)):ti,ab OR (easing NEAR/3 (restriction* OR measure* OR intervention*)):ti,ab OR ('trigger to lift':ti,ab OR 'triggers to lift':ti,ab OR 'trigger to ease':ti,ab OR 'triggers to ease':ti,ab OR 'trigger to relax':ti,ab OR 'triggers to relax':ti,ab OR 'threshold to lift':ti,ab OR 'thresholds to lift':ti,ab OR 'threshold to ease':ti,ab OR 'thresholds to ease':ti,ab OR 'threshold to relax':ti,ab OR 'thresholds to relax':ti,ab)) |

The third search, EMBASE 3, was designed to further explore the stage of most interest, including the new term of 'resurgence', as this seemed the topic most closely allied to decisions about de-escalation in the literature.

Table 3: EMBASE 3 search strategy

| **No.** | **Query** |
| --- | --- |
| #30 | resurgence:ti,ab |
| #31 | (('emergency preparedness'/exp OR 'preparedness'/exp OR 'disaster planning'/exp) OR (emergency NEAR/3 (prepar* OR respon* OR plan*)):ti,ab OR (pandemic NEAR/3 (prepar* OR respon* OR plan*)):ti,ab OR (outbreak NEAR/3 (prepar* OR respon* OR plan*)):ti,ab OR 'after action review':ti,ab OR 'critical incident review':ti,ab OR 'organization and management'/exp/mj) AND resurgence:ti,ab |
| #32 | ('exit strategy':ti,ab OR ('de-escalat*':ti,ab OR 'de escalat*':ti,ab OR 'deescalat*':ti,ab) OR ('de-isolat*':ti,ab OR 'de isolat*':ti,ab OR deisolat*:ti,ab) OR ('re-open*':ti,ab OR 're open*':ti,ab OR reopen*:ti,ab) OR (relax* NEAR/3 (restriction* OR measure* OR intervention*)):ti,ab OR (lift* NEAR/3 (restriction* OR measure* OR intervention*)):ti,ab OR (ease NEAR/3 (restriction* OR measure* OR intervention*)):ti,ab OR (easing NEAR/3 (restriction* OR measure* OR intervention*)):ti,ab OR ('trigger to lift':ti,ab OR 'triggers to lift':ti,ab OR 'trigger to ease':ti,ab OR 'triggers to ease':ti,ab OR 'trigger to relax':ti,ab OR 'triggers to relax':ti,ab OR 'threshold to lift':ti,ab OR 'thresholds to lift':ti,ab OR 'threshold to ease':ti,ab OR 'thresholds to ease':ti,ab OR 'threshold to relax':ti,ab OR 'thresholds to relax':ti,ab)) AND resurgence:ti,ab |
| #33 | #31 or #32 |
| #34 second search | #33 AND [1-4-2020]/sd |

#### Scopus search strategy

The Scopus search strategy was a translation of the EMBASE 3 search strategy, as this was the most refined version of the search strategy. As the search output was of a reasonable size for sifting, the term resurgence was not included, as this would have narrowed the output. Scopus was selected because of its multidisciplinary nature and coverage of topic areas not included in Embase.com. It does not include a structured thesaurus in the same way as Embase, therefore our search focused on title and abstract. The Scopus search was carried out on April 9th 2020.

Table 4: Scopus search strategy

| **No.** | **Query** |
| --- | --- |
| #1 | ( ( emergency OR pandemic  OR outbreak ) W/3 ( prepar*  OR respon* OR plan* ) ) AND ( exit  OR de-escalat* OR de-isolat* OR re-open  OR relax* OR lift* OR ease OR trigger OR  threshold ) |
| #2 | ( ( pandemic  OR outbreak )  W/3 ( prepar* OR  respon* OR plan* ) )  AND ( exit OR de-escalat*  OR de-isolat* OR re-open OR  relax* OR lift* OR ease OR trigger  OR threshold ) |
| #3 | ( ( pandemic  OR outbreak )  W/3 ( prepar* OR  respon* OR plan* ) )  AND ( exit OR de-escalat*  OR de-isolat* OR re-open OR  relax* OR lift* OR ease OR trigger  OR threshold ) AND ( LIMIT-TO ( PUBYEAR ,  2020 ) OR LIMIT-TO ( PUBYEAR , 2019 ) OR LIMIT-TO ( PUBYEAR ,  2018 ) OR LIMIT-TO ( PUBYEAR , 2017 ) OR LIMIT-TO ( PUBYEAR , 2016 )  OR LIMIT-TO ( PUBYEAR , 2015 ) OR LIMIT-TO ( PUBYEAR , 2014 ) OR LIMIT-TO ( PUBYEAR ,  2013 ) OR LIMIT-TO ( PUBYEAR , 2012 ) OR LIMIT-TO ( PUBYEAR , 2011 ) OR LIMIT-TO ( PUBYEAR ,  2010 ) OR LIMIT-TO ( PUBYEAR , 2009 ) OR LIMIT-TO ( PUBYEAR , 2008 ) OR LIMIT-TO ( PUBYEAR , 2007 )  OR LIMIT-TO ( PUBYEAR , 2006 ) OR LIMIT-TO ( PUBYEAR , 2005 ) OR LIMIT-TO ( PUBYEAR , 2004 ) OR LIMIT-TO ( PUBYEAR ,  2003 ) OR LIMIT-TO ( PUBYEAR , 2002 ) OR LIMIT-TO ( PUBYEAR , 2001 ) OR LIMIT-TO ( PUBYEAR , 2000 ) ) |
| #3 | TITLE-ABS ( ( pandemic  OR outbreak ) W/3 ( prepar*  OR respon* OR plan* ) ) AND ( exit  OR de-escalat* OR de-isolat* OR re-open  OR relax* OR lift* OR ease OR trigger OR  threshold ) AND ( LIMIT-TO ( PUBYEAR , 2020 ) OR  LIMIT-TO ( PUBYEAR , 2019 ) OR LIMIT-TO ( PUBYEAR ,  2018 ) OR LIMIT-TO ( PUBYEAR , 2017 ) OR LIMIT-TO ( PUBYEAR ,  2016 ) OR LIMIT-TO ( PUBYEAR , 2015 ) OR LIMIT-TO ( PUBYEAR , 2014 )  OR LIMIT-TO ( PUBYEAR , 2013 ) OR LIMIT-TO ( PUBYEAR , 2012 ) OR LIMIT-TO ( PUBYEAR ,  2011 ) OR LIMIT-TO ( PUBYEAR , 2010 ) OR LIMIT-TO ( PUBYEAR , 2009 ) OR LIMIT-TO ( PUBYEAR ,  2008 ) OR LIMIT-TO ( PUBYEAR , 2007 ) OR LIMIT-TO ( PUBYEAR , 2006 ) OR LIMIT-TO ( PUBYEAR , 2005 )  OR LIMIT-TO ( PUBYEAR , 2004 ) OR LIMIT-TO ( PUBYEAR , 2003 ) OR LIMIT-TO ( PUBYEAR , 2002 ) OR LIMIT-TO ( PUBYEAR ,  2001 ) OR LIMIT-TO ( PUBYEAR , 2000 ) ) |
| #4 second search | TITLE-ABS (( pandemic  OR outbreak ) W/3 ( prepar*  OR respon* OR plan* ) ) AND ( exit  OR de-escalat* OR de-isolat* OR re-open  OR relax* OR lift* OR ease OR trigger OR  threshold ) AND ( LIMIT-TO ( PUBYEAR , 2020 ) OR  LIMIT-TO ( PUBYEAR , 2021)) |
