# Supplementary material for: De-escalation strategies for non-pharmaceutical interventions following infectious disease outbreaks: a rapid review and a proposed dynamic de-escalation framework
Source: Global Health. 2021 Sep 16;17:106. doi: 10.1186/s12992-021-00743-y (PMC8444163; doi:10.1186/s12992-021-00743-y)
Supplement: Supplementary file 2 — Additional file 2: [file 12992_2021_743_MOESM2_ESM.docx]

#### "This supplementary material is hosted by *Globalization and Health* as supporting information alongside the article [De-escalation strategies for non-pharmaceutical interventions following infectious disease outbreaks: a rapid review and a proposed dynamic de-escalation framework], on behalf of the authors, who remain responsible for the accuracy and appropriateness of the content. The same standards for ethics, copyright, attributions and permissions as for the article apply. Supplements are not edited by *Globalization and Health* and the journal is not responsible for the maintenance of any links or email addresses provided therein."

Table 1: Documents included in final analysis by type and setting

| **Document type** | **Citation** | **Geography** | **Year(s)** | **Type of hazard, risk, emergency or event** |
| --- | --- | --- | --- | --- |
| Commentary | Christakis, D.A. | US | 2020 | Covid-19 |
| Commentary | Dzobo, M. et al. | Zimbabwe | 2020 | Covid-19 |
| Commentary | Eyawo, O. et al. | LMIC | 2021 | Covid-19 |
| Commentary | Gilbert, M. et al. | Global | 2020 | Covid-19 |
| Commentary | Godwin A. | UK | 2021 | Covid-19 |
| Commentary | Leung, G. | Global | 2020 | Covid-19 |
| Commentary | McKee, M. | Europe | 2020 | Covid-19 |
| Commentary | Petersen, E. et al. | Global | 2020 | Covid-19 |
| Commentary | Pollok, A. | UK | 2020 | Covid-19 |
| Commentary | Romer, P. et al. | US | 2020 | Covid-19 |
| Commentary | Sheikh, A. et al. | UK | 2020 | Covid-19 |
| Commentary | Searchinger, T. | US | 2020 | Covid-19 |
| Commentary | Wu, Y et al. | Hong Kong | 2020 | Covid-19 |
| Guidelines | ECDC | Europe | 2020 | Covid-19 |
| Guidelines | ECDC | Europe | 2020 | Covid-19 |
| Modelling study | Araz, O.M. et al. | Global | 2012 | Infectious disease outbreak |
| Modelling study | Chowdhury, R. et al. | Global | 2020 | Covid-19 |
| Modelling study | De Vlas, S.J. | Global | 2020 | Covid-19 |
| Modelling study | Di Domenico, L. et al. | Ile de France | 2020 | Covid-19 |
| Modelling study | Ferguson, N.M. et al. | UK/US | 2020 | Covid-19 |
| Modelling study | Glass, K. et al. | Global | 2007 | Influenza |
| Modelling study | Hatef, E. et al. | US | 2021 | Covid-19 |
| Modelling study | Kissler, S. et al. | US | 2020 | Covid-19 |
| Modelling study | Neufeld, Z. et al. | Global | 2020 | Covid-19 |
| Modelling study | Rawson, T. et al. | UK | 2020 | Covid-19 |
| Modelling study | Schmitt, F-J. | Germany | 2020 | Covid-19 |
| Plan | CDC | US | 2009 | Influenza |
| Plan | Department of Health | Australia | 2019 | Influenza |
| Plan | Gottlieb, S. et al. | US | 2020 | Covid-19 |
| Plan | Emanuel, Z. et al. | US | 2020 | Covid-19 |
| Plan | HHS | US | 2005 | Influenza |
| Plan | New Zealand Ministry of Health | New Zealand | 2017 | Influenza |
| Plan | WHO | Global | 04/2020 | Covid-19 |
| Plan | WHO | Global | 11/2020 | Covid- 19 |
| Policy paper | Abele-Brehm, A. et al. | Germany | 2020 | Covid-19 |
| Policy paper | Allen, D. et al. | US | 2020 | Covid-19 |
| Review | D’Angelo, D. et al. | Global | 2021 | Covid-19 |
| Observational Study | Somekh, I. et al. | Israel | 2021 | Covid-19 |
| Observational Study | Teixeira da Silva et al. | Italy | 2021 | Covid-19 |

Table 2: Summary of main non-pharmaceutical interventions

| **Policy** | **Description** |
| --- | --- |
| Case isolation | Isolation of symptomatic cases in hospital or their home. Reduce non-household contacts. |
| Close contact isolation | Isolation of asymptomatic potentially exposed individuals at home or in a healthcare facility, monitoring for symptoms. |
| Closure | Closure of an institution or business, e.g. schools, workplaces. |
| Community-wide containment | Essential contact to ensure vital supplies only. Also referred to as “lockdown”. |
| Contact tracing | Identification and follow-up of persons who may have come into contact with an infected person |
| Environmental measures | Routine cleaning of frequently used surfaces and objects to reduce transmission. |
| Movement restriction | Limitation on the movements of a person who has or is suspected of having an infection |
| Personal protective measures | Measures to reduce personal risk of infection, such as respiratory etiquette (e.g. using tissues when coughing or sneezing), hand washing and face masks |
| Physical distancing of entire population | All households reduce contact outside of household, school or workplace |
| Physical distancing of at-risk groups | Reduce contacts outside of the household. |
| Travel-related measures | Advice or restrictions on the movement of travellers including entry or exit screening, international travel restrictions and border closures. |
